# Tuning cell behavior with nanoparticle shape

**DOI:** 10.1371/journal.pone.0240197

**Published:** 2020-11-13

**Authors:** Edoardo Scarpa, Cesare De Pace, Adrian Steve Joseph, Senio Campos de Souza, Alessandro Poma, Eva Liatsi-Douvitsa, Claudia Contini, Valeria De Matteis, Josep Samitier Martí, Giuseppe Battaglia, Loris Rizzello

**Affiliations:** 1 Department of Chemistry, University College London, London, United Kingdom; 2 Department of Chemistry, Imperial College London, Molecular Sciences Research Hub, London, United Kingdom; 3 Department of Mathematics and Physics, University of Salento, Lecce, Italy; 4 Institute for Bioengineering of Catalonia (IBEC), The Barcelona Institute of Science and Technology, Barcelona, Spain; 5 Catalan Institution for Research and Advanced Studies (ICREA), Barcelona, Spain; 6 University of Barcelona (UB), Barcelona, Spain; 7 Networking Biomedical Research Center for Bioengineering, Biomaterials and Nanomedicine (CIBER-BBN), Madrid, Spain; 8 Institute for the Physics of Living Systems, University College London, London, United Kingdom; 9 Department of Pharmaceutical Sciences, University of Milan, Milano, Italy; Peking University, CHINA

## Abstract

We investigated how the shape of polymeric vesicles, made by the exact same material, impacts the replication activity and metabolic state of both cancer and non-cancer cell types. First, we isolated discrete geometrical structures (spheres and tubes) from a heterogeneous sample using density-gradient centrifugation. Then, we characterized the cellular internalization and the kinetics of uptake of both types of polymersomes in different cell types (either cancer or non-cancer cells). We also investigated the cellular metabolic response as a function of the shape of the structures internalized and discovered that tubular vesicles induce a significant decrease in the replication activity of cancer cells compared to spherical vesicles. We related this effect to the significant up-regulation of the tumor suppressor genes *p21* and *p53* with a concomitant activation of caspase 3/7. Finally, we demonstrated that combining the intrinsic shape-dependent effects of tubes with the delivery of doxorubicin significantly increases the cytotoxicity of the system. Our results illustrate how the geometrical conformation of nanoparticles could impact cell behavior and how this could be tuned to create novel drug delivery systems tailored to specific biomedical application.

## 1. Introduction

Deeper understanding of the interactions between nanomaterials and living systems is uncovering unforeseen cellular behaviors and paving the way for novel biomedical applications. We are now able to generate an enormous set of nano-sized structures with defined physicochemical properties [1]. However, it is important to remember that the nanoscopic nature of these structures can interfere with the physiological processes taking place within the cells at the mesoscopic level. Therefore, it is paramount to explore, and even categorize, the boundless field of interactions between nanoparticles, cellular components and molecular targets according to the physicochemical properties of the nanoparticles investigated [2–4]. In this regard, it is widely established that geometry, size, topography and topology of a given nanoscopic structure have a great impact on the intended biological application [5,6]. Viruses are a great example, having evolved around peculiar geometries (*e*.*g*. icosahedral, bullet shape and rod) in order to maximize their ability to travel the human body and enter and infect host cells [7]. Similarly, synthetic nanoparticles with different shapes display differences in circulation time [1], in the ability to extravasate from the blood stream [8], as well as in targeting efficiency [9,10]. Even at a molecular level, it is possible to favor cell uptake, and drug delivery indeed, by tuning the shape of the nanoparticles [11–13]. It is generally accepted that shapes favoring the cell membrane anchoring and the following wrapping (e.g. spherical shaper) also promote cellular internalization [14]. These features are mostly, but not exclusively, influenced by the aspect ratio of the nanoparticles [15], and by the presence of defined sharp-edges in the final morphological structure [16]. However, it could also be speculated that cells will activate different internalization mechanisms and molecular pathways according to the specific shape of the nanoparticles. Partial indications have been provided by the fact that nanoparticles of same chemistry but different shape (micelles, spheres, rods and worms) are differentially trafficked within the cells, and accumulate in distinctive cellular compartments [17]. These aspects are particularly relevant also because they could notably impact cell functionality [18]. Nevertheless, a limitation for addressing such topic comes from the significant difficulties in obtaining monodisperse nanomaterials with a narrow size and/or shape distribution [19]. In addition, there is a general lack of characterization criteria, in terms of both nanomaterials physicochemical properties and final biological effects. Despite more than a decade of research works, drawing a general principle regarding the role of nanoparticle’s shape in inducing specific molecular outcomes in cells seems a futile exercise. Nonetheless, it is worth investigating how altering the aspect-ratio of a given nanomaterial affects the biological response.

Here, we explore the biomolecular effects induced by spherical and tubular polymersomes on cancer and non-cancer cell lines. We used the pH-responsive amphiphilic diblock copolymer poly(2-(methacryloyloxy)ethyl phosphorylcholine)–poly(2-(diisopropylamino)ethyl methacrylate) (PMPC–PDPA) [20], to produce vesicles with two distinct geometrical structures (spherical or tubular). The PMPC-PDPA copolymers bestow the vesicles with three critical features, namely the ability (i) to resist non-specific protein adsorption [21], (ii) to target the endocytosis-related receptor SR-B1 [22] and once within early endosome (iii) to escape them via a pH decrease-triggered disassembly [23]. Such a combination has made this pH-sensitive copolymer widely exploited in various biomedical applications, especially for DNA, drugs, and antibodies cellular delivery, but it is also a promising candidate for tumor targeting and treatments [23–27]. We previously demonstrated that both spherical and tubular polymersomes can be purified and isolated from a mixed population [28]. Further investigations showed that tubular polymersomes display a delayed kinetic of cellular internalization compared to spherical structures [12]. Here we hypothesized that such slow kinetics of internalization of the tubular structures would create a local destabilization of the plasma membrane. This, together with the high aspect ratio of the tubes that exceeds the size of a mature endosome, would result in different intracellular fates and final cellular responses.

First, we employed the already published method for separating nanoparticles and isolating two geometrically distinct population of polymersomes (*i*.*e*., spheres and tubes), based on density gradient centrifugation [28]. This enabled us to work with well-defined samples with one shape distribution. Then, we investigated the internalization kinetics and the cytotoxicity induced by these two nano-sized structures comparing two immortalized cancer-cell lines, HeLa and FaDu, and one primary, healthy, human dermal fibroblast (HDF).

We assessed the activation of specific apoptotic pathways and quantified the replication activity of cells through the cytome assay. We also investigated the potential molecular targets of such nanostructures determining the expression of a panel of genes involved in general stress responses (*e*.*g*., oxidative stress and unfolded protein response pathway), with a focus on the tumor suppressor genes p53 and p21. Interestingly, we found out a strong correlation between the regulation of specific genes and the outcomes of the cytome assay, especially in terms of cell proliferation activity. Notably, the molecular response to nanoparticle shape was found to be cell-specific, so that cancer cells behaved differently compared to primary fibroblasts. Finally, we loaded either tubular or spherical vesicles with doxorubicin, a model chemotherapeutic compound widely used in clinic and the focus of numerous previous investigations involving nanomaterials and polymersomes [29,30]. We confirmed that doxorubicin-loaded tubular vesicles exhibit a higher toxicity towards cancer-cells compared to spherical vesicles. The data presented here shows that cells respond to the two different nanomaterial geometries by activating diverse molecular pathways. Our observations also indicate differences of response between cancer and non-cancer cell types. To the best of our knowledge, this is the first line of evidence showing that the shape of the polymersomes can be used to affect cellular behavior and increase the effect of cytotoxic drugs. These results provide new suggestions for the development of novel nanoparticle-based anticancer therapies.

## 2. Results and discussion

### 2.1 Isolation of metastable phases

**Fig 1:**
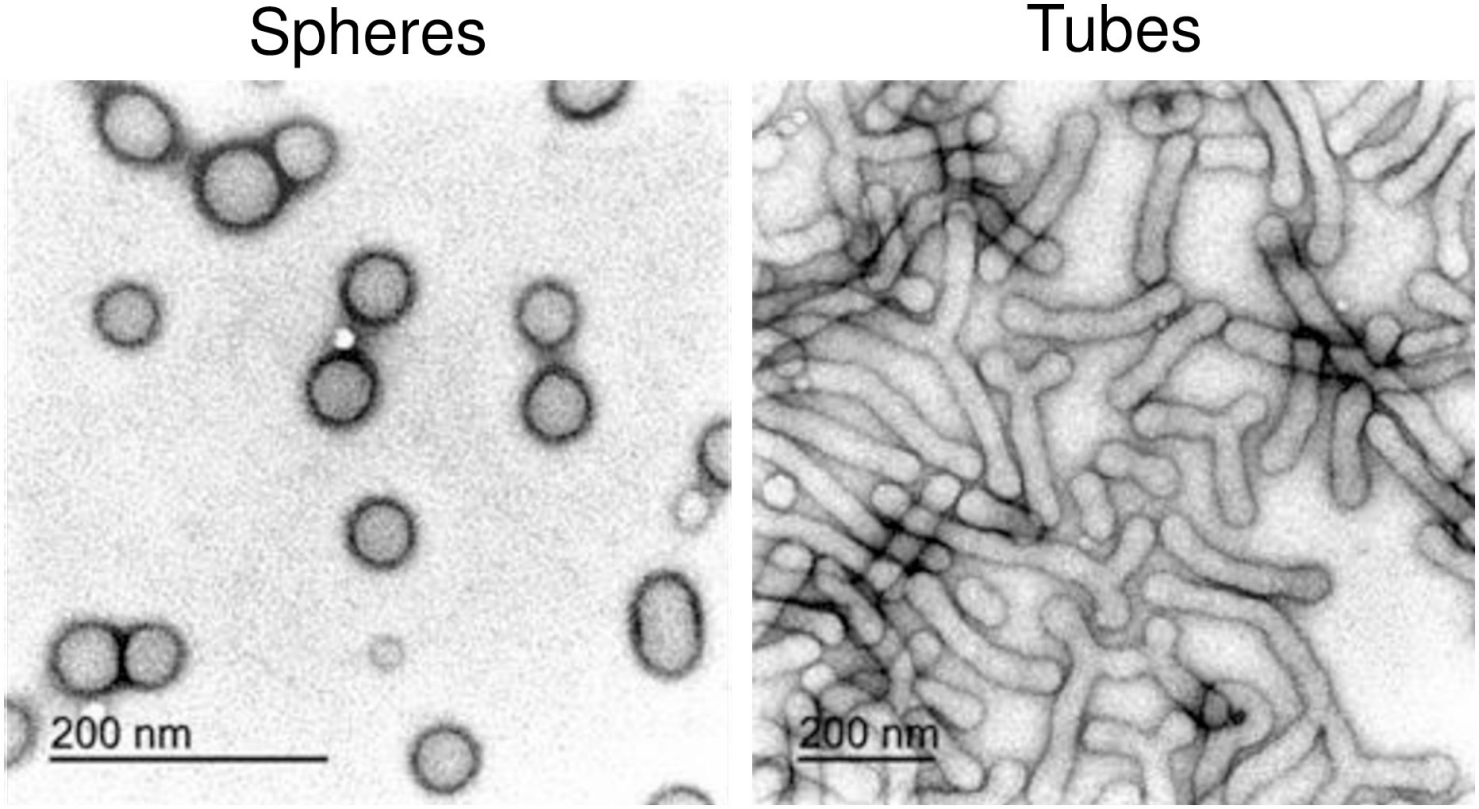
TEM characterization of spherical and tubular polymersomes. Transmission electron microscopy micrographs showing the morphologies of the polymersomes following preparation and purification using sucrose-based density gradient centrifugation.

In our experimental approach, we used the diblock copolymer poly(2-(methacryloyloxy)ethyl phosphorylcholine)–poly(2-(diisopropylamino)ethyl methacrylate) (PMPC–PDPA) to produce spherical and tubular vesicles by film rehydration, also known as top-down approach [31]. With this method, PMPC-PDPA is dissolved into a glass vial with an organic solution that is then evaporated. The film of polymer obtained is rehydrated with phosphate buffer saline (PBS) under continuous stirring for 8 weeks. During the rehydration, the diffusion of water in the dry film of block copolymer leads to the formation of self-assemblies which eventually evolve into lamellar structures first and then into vesicles [32]. However, the mixing of the lamellar structures with the water phase creates ‘finger-like’ perturbations that result in the formation of tubular vesicles [12]. We previously demonstrated that a simple post-production purification method, exploiting sucrose-based density gradient centrifugation, enables isolating spherical from tubular vesicles [28]. We believe that operating with a highly defined sample is mandatory in nanomedicine, providing the chance to correlate more precisely the physicochemical properties of a given drug delivery system to a specific biological outcome. Using this methodology, we were able to separate and characterize the two structural sub-species, which were then used to assess cellular responses (Fig 1 and S1 Fig).

### 2.2 Cellular internalization of polymeric tubes and vesicles

We previously reported that both spherical and tubular polymersomes enter the cells but with different kinetics [12]. Here we were set to understand the molecular cascade downstream of the cellular internalization.

**Fig 2:**
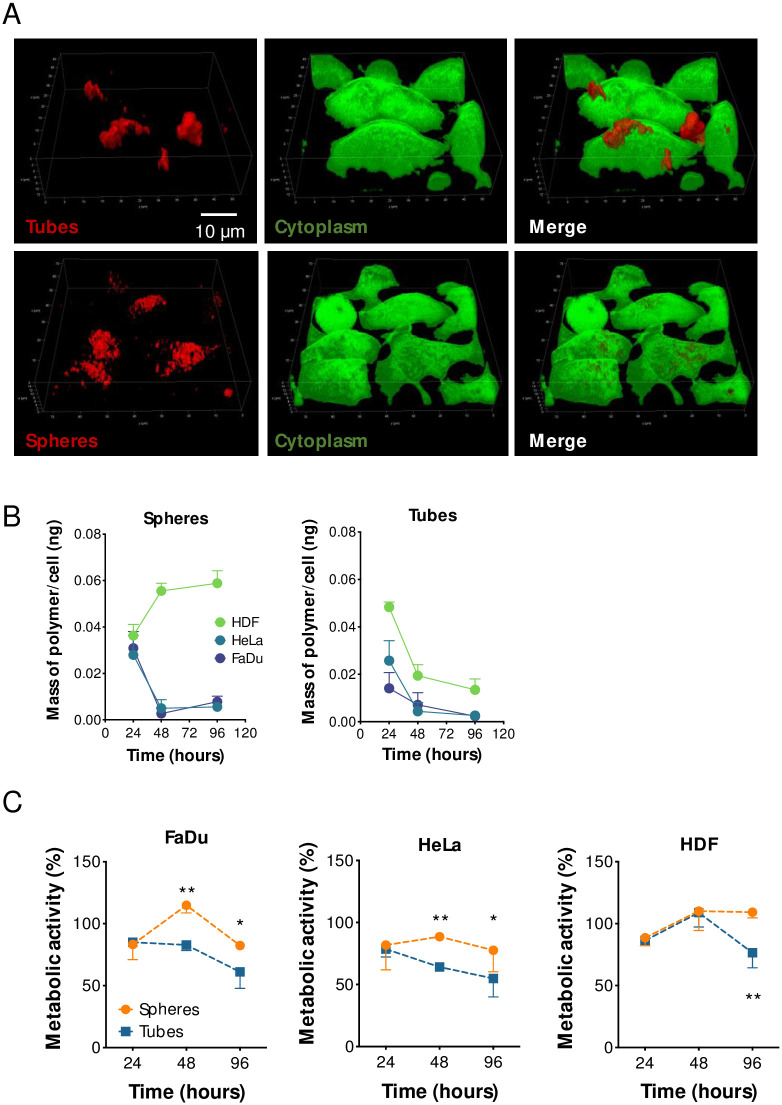
Cellular uptake of spheres and tubes. **(A)** Confocal imaging showing the complete internalization of spheres after 96 hours incubation with FaDu cells and the “frustrated” uptake of the tubes. Green is Calcein staining of the cytoplasm. **(B)** HPLC-based quantification of the mass of polymer per cell over time in either FaDu, HeLa or HDF cells. **(C)** Viability assay on FaDu, HeLa, or HDF cells incubated with spheres or tubes for 24, 48 or 96 hours. Two-way ANOVA. * = *p* < 0.05, ** = *p* < 0.01. n = 3 independent experiments.

First, we incubated three representative cell types, FaDu, HeLa and primary human dermal fibroblasts (HDF) with equal concentrations of rhodamine B-loaded spheres or tubes, and deliberately waited a prolonged time (96 hours) before imaging the cells. Despite the lengthy incubation time, we still observed remarkable differences in the spatial localization of the two structures within the cells. As demonstrated by the confocal imaging, the fluorescent spheres are widely distributed throughout the cytosol, while the tubular vesicles are found not only within the cytosol, but also associated to the cellular surface (Fig 2A and S2 Fig). This substantial dwelling on the cell membrane, that also appears to be localized in specific regions of the membrane, could be due to the high aspect-ratio of the tubular structures and it results in a delayed or “frustrated” endocytosis [12,33,34]. We speculated that this physical impairment should also result in a reduced level of cellular internalization. However, by quantifying the mass of polymer internalized after incubation with tubular structures, we observed a time-dependent decrease in concentration of polymer per cell, regardless of the cell type investigated (28, 16 and 10% decrease over 96 hours incubation for HDF, FaDu and HeLa, respectively). Spheres displayed similar trend only when in contact with cancerous cell lines (25 and 20% decrease over 96 hours incubation for FaDu and HeLa, respectively), while prolonged incubation of HDF with spheres resulted in a progressive increase in polymer per cell (Fig 2B). The heterogeneity of uptake across the cell lines is probably due to the higher replication activity of the cancer cells that results in an increased dilution of the intracellular loading as a function of cell division [35]. Contrarily, particles endocytosis in HDF, and non-cancerous cells in general, can be faster than the replication activity, and consequently the quantity of vesicles/cells tends to increase over-time [36,37]. The decreasing amount of tubes/cell as function of time is probably due to the inherent kinetics of uptake dictated by the geometrical shape of the tubes [12]. Whereby, the prolonged ‘frustrated’ endocytosis could be inhibiting the cells from internalizing further material. Considering that cells interact and process spheres and tubes in different ways, we argued whether the geometrical conformation could also lead to differential cellular responses.

We then measured cell viability after exposure to either tubes or spheres, and observed a reduction of more than 40% (*p* < 0.001) when cancer cells (FaDu and HeLa) were incubated with tubes for 48 hours compared to spheres (Fig 2C). This first set of results demonstrates that the geometrical shape of the vesicles can influence the cellular uptake and viability. Also, cancer cells appear to be particularly affected by the internalization of tubular vesicles.

### 2.3 Cytostatic activity of polymeric tubular vesicles on cancer cells

**Fig 3:**
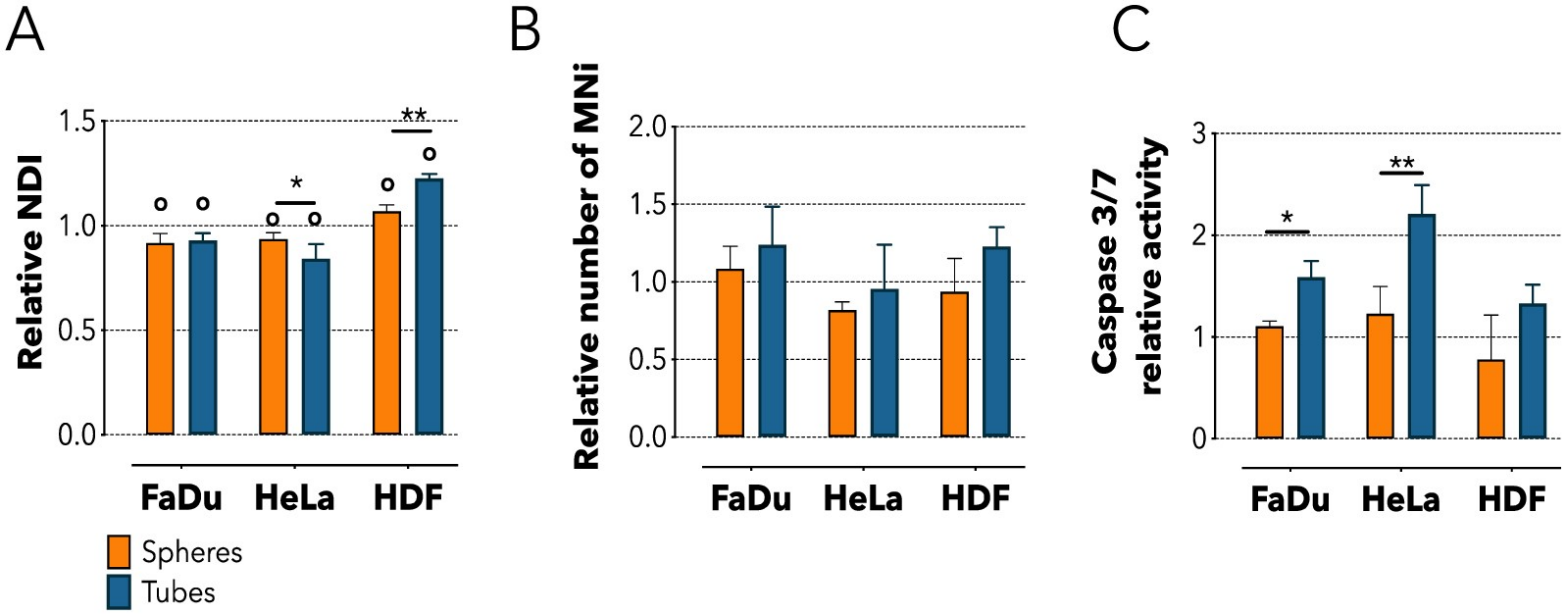
Cellular metabolic effects following spheres or tubes internalization. **(A)** Nuclear Division Index (NDI) quantification for FaDu, HeLa and HDF cells after 24 hours incubation with spheres or tubes. **(B)** Micronucleus assay (MNi) for addressing the presence of DNA damage after 24 hours incubation with spheres or tubes. **(C)** Caspase 3/7 assay for analyzing the activation of extrinsic apoptosis following 24 hours incubation with spheres or tubes. Two-way ANOVA. * = *p* < 0.05, ** = *p* < 0.01, o = statically different compared to the untreated control. n = 3 independent experiments.

We then investigated the underlining effects triggered by the internalization of polymeric tubes by cancer cells. First, we assessed whether tubes and spheres interact differently with the cellular replication machinery using the cytokinesis-block micronucleus cytome assay [38]. Here, cells are treated with the mycotoxin cytochalasin-B (Cyt-B) which impedes the final stage of cytokinesis inhibiting the microfilament assembly [38,39]. This method assesses mitosis and cytostasis by quantifying the nuclear division index (NDI), as well as chromosome damaging by calculating the number of micronuclei (MNi) formed. Using an *ad hoc* automated script, we analyzed images of more than 6000 cells (S3 Fig) and found that 24 hours treatment with tubes or spheres significantly reduced the NDI of FaDu cells compared to the untreated control (*p* < 0.05). Similarly, incubating HeLa cells with tubes induced a 1.2 folds reduction in NDI compared to untreated control (*p* < 0.01), and also a significant reduction compared to spheres (*p* < 0.05, Fig 3A). The cytostatic effect exerted by the tubes on cancer cell lines was completely reversed in HDF. Here these structures increased the NDI of more than 16% compared to control (*p* < 0.0001), and ~15% compared to the spheres (*p* < 0.01) (Fig 3A). Note that despite affecting the mitosis of the cancer cells investigated, neither spheres nor tubes resulted to be genotoxic for the cells (Fig 3B). Overall, we observed that in cancer cell both spheres and tubes induced a significant decrease in the relative NDI, thus reflecting a tendency to slow down their replication activity. However, the effect mediated by the tubes appeared to be more marked in HeLa cells. Contrarily, the treatment with tubes induced an increase in the replication activity of primary fibroblasts potentially due to cellular stress. The change in the NDI values highlights the interaction occurring between the replication machinery of cells and nanostructures that, in turn, is strongly influenced by the geometrical conformation of such structures. Also, HDF had the opposite behavior compared to HeLa or FaDu upon incubation with tubes, underlining how cells of different nature (tumorigenic and non) activate differential cellular pathways in response the to the same physicochemical cue. These molecular pathways do not lead nor are involved with DNA damage, as showed by the MNi analysis, but could trigger apoptosis.

Having determined that tubes are cytostatic once in contact with cancerous cells, we explored whether this effect could be related to the activation of caspase proteins. We investigated the activity of caspase 3/7 that become functional upon the binding of exogenous ligands to cell death receptors. We observed a significant activation of caspase 3/7 following incubation of either FaDu or HeLa cells with tubes, while spheres were essentially inert. On the other hand, HDF cells did not exhibit any caspase activation in any of the conditions tested (Fig 3C). The data indicate that the internalization of tubes by HeLa or FaDu cells induces the activation of the extrinsic pathway of apoptosis mediated by the slight activation of caspase 3/7, while spheres had not detectable effects on cells [40]. On the other side, we did not observe the activation of caspase 9, confirming that intrinsic apoptosis (triggered by DNA damage) does not occur. This result suggests that the apoptotic trigger is likely given by the interaction between tubes and the extracellular death receptors.

**Fig 4:**
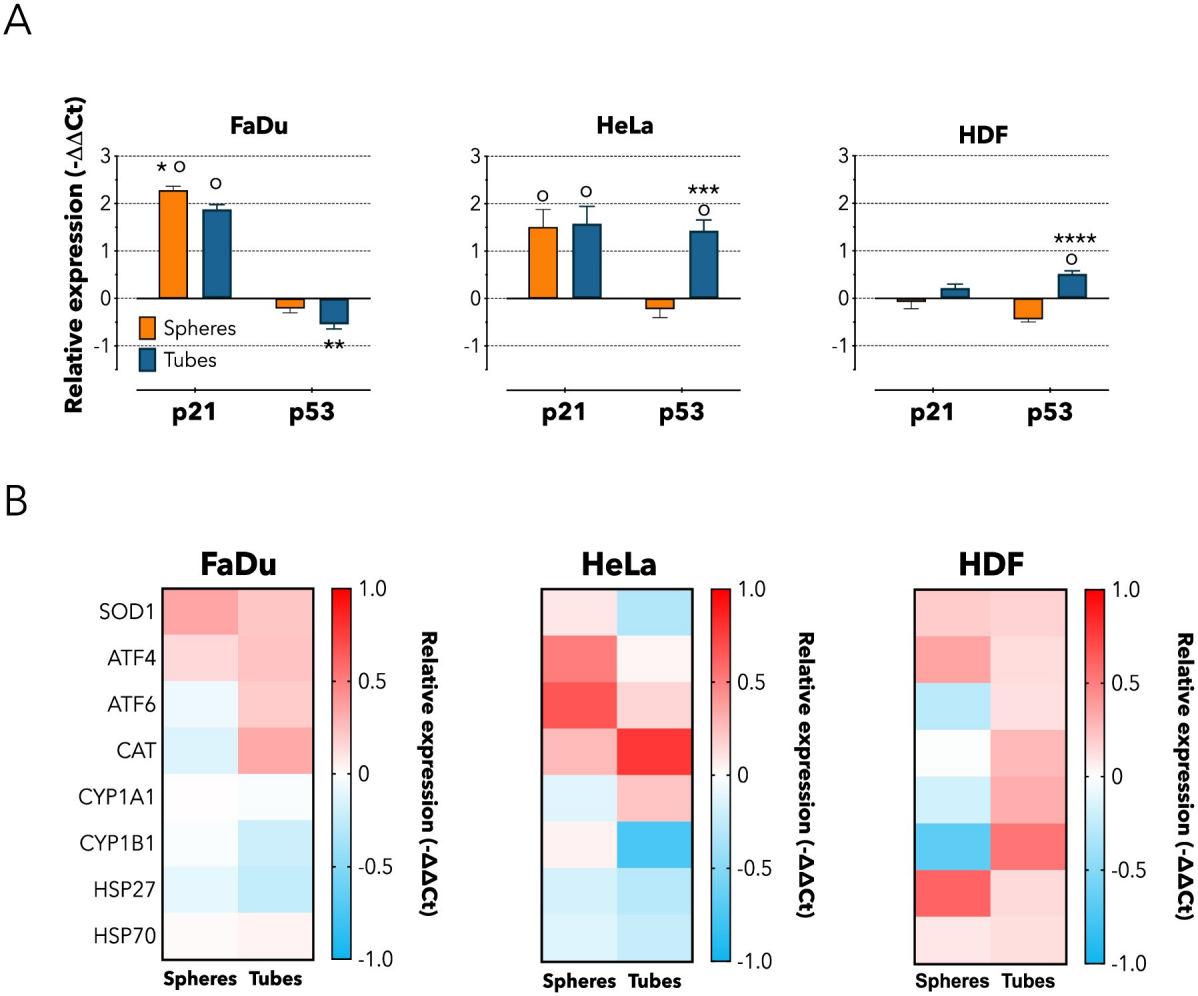
Cellular metabolic effects following spheres or tubes internalization. (A) Real Time qPCR for quantifying the expression of genes involved in replication activity (*p21*, *p53*) following 24 hours incubation with spheres or tubes. Two-way ANOVA. * = *p* < 0.05, ** = *p* < 0.01, *** = *p* < 0.001, **** = *p* < 0.0001; o = statically different compared to the untreated control. n = 3 independent experiments. (B) Heat map showing real time qPCR quantification of genes involved in oxidative stress (SOD1, CAT), detoxification metabolism (CYP1A1, CYP1B1), Unfolded Protein Response—UPR (ATF4, ATF6), and general shock (HSP27, HSP70). Cells were incubated for following 24 hours incubation with spheres or tubes. n = 3 independent experiments.

The activation of the caspase pathway is linked to the up-regulation of executioners at genetic and molecular level, hence we analyzed the expression of genes involved in the cell cycle, oxidative stress, detoxification metabolism, unfolded protein response and general cellular shock. First, we investigated *p53* and *p21*. The former responds to stress signals by (i) arresting cells cycle, and by (ii) promoting cell senescence and apoptosis [41]. Whilst, the cyclin-dependent kinase inhibitor *p21* acts as both sensor and actuator of multiple anti-proliferative signals [42]. We observed a significant up-regulation of *p21* in cancer cells following incubation with either tubes or spheres compared to untreated control. However, tubes also affected the expression of *p53* in a differential way depending on the cell types. Compared to both untreated control and spheres, *p53* was down-regulated in FaDu cells (*p* < 0.01) and up-regulated in both HeLa (*p* < 0.001) and HDF cells (*p* < 0.0001, Fig 4A). This differential expression is intriguing, but it should be considered that usually *p21* and *p53* are part of close-related pathways, so that they influence each other expression. However, there are several evidences that *p21* and *p53* may also act in independent ways [41–43]. Hence, our data suggests that the treatment with either spherical or tubular vesicles lead to the activation of the alternative pathway of *p21*, independently on *p53*. This different regulation could be mediated by the engagement of the scavenger receptor class B-Type I (SRBI) on the cell membrane by the PMPC polymer [22], which could activate alternative downstream molecular cascades. However, further experiments would be needed in order to confirm this speculation.

Moreover, following incubation with tubes, we observed an increased level of oxidative stress and signs of metabolic detoxification, indicated by the enhanced expression of CAT (*p* < 0.05), and the down-regulation of CYP1B1. However, the expression of other stress-related genes was not significantly altered (Fig 4B). Interestingly, the expression of CAT, CYP1A1 and CYP1B1 in HDF was also up-regulated following incubation with tubular vesicles, while it was down-regulated upon treatment with spheres. It could be speculated that the enhanced expression of these genes might be the prelude to the reduced metabolic activity observed after 48 hours of treatment upon incubation with tubes. It is also worth noting how incubation with either type of vesicle results in a down-regulation of HSP27 in cancer cell lines and in an up-regulation in HDF cells. As this gene is involved in chemotherapeutic resistance and its inhibition enhances the effects of different therapies [44], it could be interesting to exploit this effect in future investigations.

This set of experiments confirmed that spheres up-regulated *p21* and tubes differentially affect cancer cells, and such effect is mediated by the activation of cell death-related signaling and induction of oxidative stress within the cells.

### 2.4 Enhanced cytotoxic effect of polymeric tubular vesicles on cancer cells

**Fig 5:**
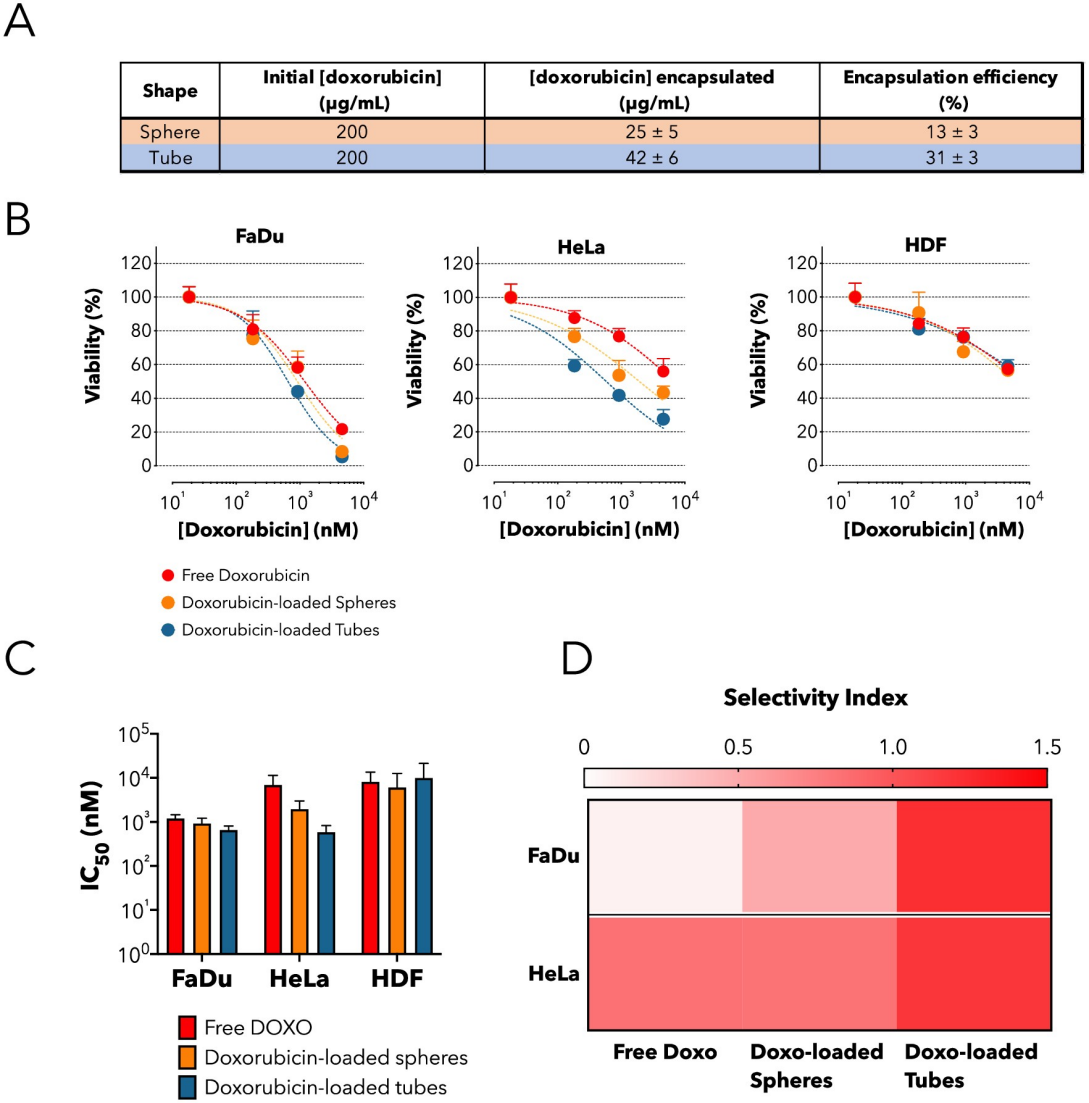
Enhanced cytotoxic activity of doxorubicin-loaded tubes. **(A)** Table summarizing the initial mass of doxorubicin solubilized with the block co-polymer, the final concentration encapsulated as measured by HPLC and the overall efficiency of encapsulation. **(B)** Cell viability test on HDF, HeLa and FaDu cells following 48 hours incubation with increasing extracellular concentrations of either free doxorubicin or doxorubicin loaded in spheres or tubes. **(C)** IC50 values (nM) for the free (green), spheres-encapsulated (orange) and tubes-encapsulated (blue) DOXO. **(D)** Heat-map showing the selectivity index (SI) of free doxorubicin, doxorubicin-loaded spheres, or doxorubicin-loaded tubes towards FaDu or HeLa cells. Note that SI is the log ratio between the IC50 in non-cancer and in cancer cells (HDF as reference).

Finally, we assessed whether negative effects induced by the incubation of tubes with cancer cells could be exploited to augment the cytotoxicity of a given chemotherapeutic molecule. As a proof of concept, we loaded doxorubicin in both spheres and tubes and we calculated the final concentration of drug encapsulated by HPLC (Fig 5A), following a purification step to remove any excess of unencapsulated drug present in solution. Finally, we measured cell viability after 48 hours of incubation time with either free doxorubicin or doxorubicin-loaded spheres or tubes. Overall, doxorubicin-loaded tubes were more cytotoxic than either spheres or free doxorubicin in both cancer cell lines; although to a different extent in the two cell types (Fig 5B). In FaDu cells, loaded-tubes induced a limited but evident reduction of the inhibitory concentration 50 (IC_50_) of 1.83- and 1.4-fold compared to free doxorubicin and spheres, respectively (IC_50_: tubes = 651 nM, spheres = 913 nM and free doxorubicin = 1190 nM) (Fig 5C). While, tubes were even more effective in Hela cells where the reduction in IC50 was 11.79-fold compared to free doxorubicin and 3.32-fold compared to spheres (Fig 5C). No significant difference was observed among tubes, spheres or free doxorubicin upon incubation with HDF cells (Fig 5B and 5C). To quantify the ability of doxorubicin-loaded spheres and tubes to selectively target cancer cells, we determined the selectivity index (SI). This is calculated as the log of the ratio between the IC50 measured for non-cancer cells (HDF) and the one measured for cancer cells (FaDu or HeLa) (SI = log (IC50 non-cancer cell/IC50 cancer cell)). The SI values reported in the heat-map demonstrate that overall loaded-tubes have a higher efficiency of action (more than 10 times) in cancer-cells compared to either free doxorubicin or loaded-spheres (Fig 5D). This set of results confirms that the combination cytostatic effect induced by the tubes with the cytotoxic activity of doxorubicin can enhance the therapeutic effect.

### Conclusions

Understanding the interactions occurring between nanomaterials and living systems is a critical aspect in the design of biomedical devices with active functional properties. Being in the same size range of proteins, nanoparticles are effectively able to interact with various molecular components of the cells, with the consequent possibility of non-specifically affecting cellular behavior. It is indeed necessary to deeply understand the way nanoparticles tune the physiology of cells. To this respect, the physicochemical properties of nanoparticles are important parameters to be considered in the process of biointeractions. In this work, we investigated the interactions between two differently shaped polymersomes (i.e. spherical and tubular vesicles), with cancer and non-cancer cells. We observed that tubular structures interact differently with cells; and cancer cells are particularly affected by such geometry. Although the investigation of only two geometrical shapes and three different cell types prevents us from drawing a general conclusion, these findings demonstrate that nanoparticle shape plays a pivotal role in the interactions with cells and severely affects cellular behavior. Nonetheless, the results reported here also open to the opportunity of properly tailoring the physicochemical properties of the nanoparticles according to the biological application.

## 3. Materials and methods

### 3.1 Preparation of PMPC_25_-PDPA_70_ structures

PMPC_25_-PDPA_70_, where 25 and 70 represent the degree of polymerization, was prepared by atom-transfer radical polymerization (ATRP) [45]. In a typical ATRP procedure, a 100 mL round bottom flask equipped with a magnetic stir bar and a rubber septum was loaded with 2-methacryloyloxyethyl phosphorylcholine (MPC, 5 g, 16.9 mmol), 2-(4-morpholino)ethyl 2-bromoisobutyrate (ME-Br) initiator (189 mg, 0.7 mmol) and 6 mL ethanol, and this solution was deoxygenated by purging N_2_ for 1 h under stirring at r.t. Then, 2,2'-bipyridine (bpy) ligand (212 mg, 1.4 mmol) and Cu(I)Br (97 mg, 0.7 mmol) were added as solids whilst maintaining the flask under a mild positive N_2_ pressure. The [MPC]:[ME-Br]:[CuBr]:[bpy] relative molar ratios were 25:1:1:2. The reaction was carried out under a N_2_ atmosphere at 30°C. After 90 minutes (MPC conversion >99%), a solution of 2-(diisopropylamino)ethyl methacrylate (DPA, 12.3 g, 57.6 mmol) in ethanol (15 mL), previously deoxygenated by purging N_2_ for 1 h at r.t., was injected into the flask. After 48 h, the reaction solution was opened to the air, diluted by addition of ethanol (≈200 mL) and left stirring for 1 h. The solution was then passed through a silica column to remove the copper catalyst. After this step, the filtrate was concentrated by rotary evaporation and dialysed using a 1 kDa MWCO dialysis membrane (Spectrum Labs, Netherland) against chloroform/methanol 2:1 (v/v) (2 × 500 mL), methanol (2 × 500 mL), and double-distilled water (4 × 2 L). At least 8 h passed between changes. After dialysis the copolymer was isolated by freeze-drying and characterised by ^1^H-NMR spectroscopy performed on an Avance III 600 spectrometer from Bruker (Billerica, USA), and gel permeation chromatography performed on a GPCMax equipped with an RI detector from Malvern Technologies (Greater Malvern, UK) with acidic water (0.25 vol% TFA in water) as solvent on a Novamax column (including guard column) from PSS Polymers (Mainz, Germany). The GPC chromatogram and NMR of the polymers produces is reported in S4 Fig.

### 3.2 Sucrose density gradient for shape-dependent NPs purification

For the purification of polymersomes by shape, we exploited a method developed in our previous work. Briefly, we used solutions of increasing sucrose concentration, dissolved in PBS, namely 5, 10, 15, 20, and 25% w/v. Aliquots of 200 μL for each solution were gently layered from the most dense to the least dense within a 1.5 mL micro-centrifuge tube, while avoiding random dispersion of each aliquot. Finally, 150 μL of solution containing a mixture of rhodamine-labelled vesicles and tubes, prepared by film rehydration, was deposited as the final top layer, and the micro-centrifuge tube was centrifuged at 20000 g for 2 hours. After centrifugation, 20 μL of each layer was collected and analyzed by TEM. Spherical polymersomes were also characterized by dynamic light scattering (DLS) using a Zetasizer Nano Zs (Malvern Ltd.) at a polymersome concentration of 0.25 mg/mL.

### 3.3 TEM characterizations

TEM analysis was performed using a JEOL 2100 operating at 200 kV equipped with a CCD camera Orius SC2001 from Gatan. Copper grids were glow discharged and the sample was adsorbed onto the grid. The sample was then stained with 0.75 wt% phosphotungstic acid (PTA) raised to pH 7.4 with NaOH. The images of tubes and spheres were analyzed by ImageJ 1.47 software (https://imagej.nih.gov/ij/) and used to investigate the morphometric parameters of the nanoparticles. 100 random nanoparticles were measured to obtain: mean size, area, perimeter, circularity and aspect ratio. Note that circularity values near 1 indicate a perfect cycle, whereas close to 0 indicate a high sharpness degree.

### 3.4 Cell cultures

Primary human dermal fibroblasts (HDF), ovarian cancer cells (HeLa), and oral carcinoma (FaDu) cells were purchased from ATCC^®^. HDF, HeLa, and FaDu cells were cultured and maintained using Dulbecco’s Modified Eagle Medium (DMEM) (Sigma-Aldrich) containing: 10% (v/v) fetal calf serum, 2 mM L-glutamine, 100 mg/ml streptomycin and 100 IU/ml penicillin (Sigma-Aldrich-Aldrich). Cells were cultured at 37°C/95% air/5% CO_2_. Cells were periodically sub-cultured using Trypsin-EDTA solution 0.25% (Sigma-Aldrich) for the detachment process and centrifuged at 2000 rpm for 5 min for the pellet collection.

### 3.5 Viability and metabolic assays

The RealTime-Glo (Promega) was used as viability test. Cells were seeded at a density of 5 x 10^3^ cells/well in a white 96 well plate. After 24 hours, cells were incubated with a concentration of 0.1 mg/ml of either tubes or spheres for 24 hours. Then, cell viability was assessed following manufactures instructions by quantifying relative viability as a function of the luminescent signal produced. Luminescence was measured with a Spark plate-reader (TECAN). For the metabolic assay, the Thiazolyl Blue Tetrazolium Blue (MTT, Sigma-Aldrich) method was used. Briefly, cells were seeded over night at a density of 5 x 10^3^ cells/well in a 96 well plate. Increasing concentrations of spheres and tubes were then added to the growth media, namely 0.5, and 1 mg/mL, for periods of 24, 48, and 96 hours. The medium growth was then removed, and an acidified solution of isopropanol was added to dissolve the water insoluble MTT formazan crystals formed. The solubilized blue crystals were measured at 570 nm using a plate reader (ELx800, BioTek). For the Trypan blue-based proliferation assay, cells were seeded at a density of 8 x 10^3^ cells/well in a six well plate, and then incubated with both spheres and tubes at a concentration of 0.5 mg/mL for 24, 48, and 96 hours. Cells were then detached with a Trypsin-EDTA solution 0.25%, and counted with an automated cell counter (TC20, Bio-Rad).

### 3.6 HPLC quantification

For the uptake quantification by HPLC, cells were seeded at a density of 8 x 10^3^ cells per well in a six-well plate and incubated with Rhodamine-labelled spheres or tubes (0.1 mg/mL final concentration). Cell were then lysed in acidified PBS (pH 2) and stored at -80°C for 48 hours to promote lysis. The lysate was then centrifuged at 20,000 g for 1 hour and the supernatant was collected for the HPLC analysis (UltiMate 3000 Standard LC Systems, Thermo Scientific).

### 3.7 Cytome assays and confocal and imaging analyses

For the evaluation of the nuclear division index (NDI) and micronuclei (MNi), cells were initially seeded in glass bottom dishes (35 mm diameter-IBIDI^®^) at a density of 8 x 10^3^ cells per well, grown O.N. in complete medium, starved for 24 hours to synchronize the cell cycle. Cells were incubated with H_2_O_2_ (positive control), spherical polymersomes, and tubes for 24 hours, washed three times with PBS (5 minutes) and incubated with cytochalasin B (Sigma-Aldrich) for 24 hours to avoid the cytoplasm division. Then, all cells were washed with PBS, fixed with formaldehyde 3.7% (v/v) for 10 minutes, permeated with 0.1% (v/v) Triton X-100 in PBS for 10 minutes, and stained with Hoechst 33258 (Sigma-Aldrich) and SYTO^®^ 9 (Sigma-Aldrich) for nuclear and cytoplasm staining, respectively. For the uptake imaging, cells were seeded in glass bottom dishes, incubated with either spheres or tubes, and finally stained with calcein using the manufacturer instructions (Life Technologies) for vital staining. Cell imaging for both NDI/MNi and uptake was carried out with a confocal microscope (Leica TCS SP8), and imaging quantification was carried out with an ad hoc designed Matlab script. For the scoring of NDI and MNi, we adopted the Fenech guidelines.

### 3.8 Reverse transcription polymerase chain reaction (RT-PCR), and PCR assays

Cultured cells, incubated for 24 hours with spheres and tubes (0.5 mg/mL), were lysed and total RNA was collected by using RNeasy Mini Kit (Qiagen). RNA concentration was measured with NanoDrop spectrophotometer (Thermo). Complementary DNA (cDNA) was synthesized from every 1 μg of total mRNA in 20 μL volume per tube with QuantiTect Rev. Transcription Kit (Qiagen). The samples were then run in a standard agarose gel (1%) for RNA quality control check. For the PCR analyses, GAPDH and ACTB were used as reference genes. Quantitative analysis was assessed with QuantiTect SYBR Green RT-qPCR Kit (Qiagen). The amplification process was done in 20 μL/tube, using the following steps: 95°C for 5 min to make active the DNA Polymerase, followed by 40 cycles of 95°C (10 s) for denaturation, and 60°C (30 s) for combined annealing and extension for all primers. Melting curve was also acquired, to analyze the sample quality, from 55°C to 99°C, by increasing of 1°C/min. Data were analyzed via ΔΔCt value. 2-ΔΔCt was calculated as follows: ΔCt = CtKi67- Ct GAPDH; ΔΔCt = ΔCt(treated) -ΔCt(control). The genes expression was analyzed using the following list of primers:

**Table:** 

Gene name	Primers
ATF6	For: CTGTTACCAGCTACCACCCA
	Rev: GGAGCCAAAGAAGGTGTTGG
ATF4	For: CCGCAACATGACCGAAATGA
	Rev: CTTGCTGTTGTTGGAGGGAC
CAT	For: CCTGAGAGAGTTGTGCATGC
	Rev: TTTCACTGCAAACCCACGAG
SOD1	For: GGAGACTTGGGCAATGTGAC
	Rev: CACAAGCCAAACGACTTCCA
CYP1A1	For: GAAGCAGCTGGATGAGAACG
	Rev: GACCTGCCAATCACTGTGTC
CYP1B1	For: CTCGAGTGCAGGCAGAATTG
	Rev: TCCTTGGGAATGTGGTAGCC
HSP70	For: CCTCAGTCTGATGGCTCCAG
	Rev: CTCCTGGTCCACTTGCATCT
HSP27	For: CCAAGTTTCCTCCTCCCTGT
	Rev: CTTTACTTGGCGGCAGTCTC
p53	For: TGGCCATCTACAAGCAGTCA
	Rev: GGTACAGTCAGAGCCAACCT
p21	For: GTGAGCGATGGAACTTCGAC
	Rev: CAGGTCCACATGGTCTTCCT

### 3.9 Apoptosis assays

For the characterization of intrinsic and extrinsic apoptosis, the luminesce-based Caspase-Glo^®^ 3/7 Assay Systems (Promega) and the Caspase-Glo^®^ 9 Assay Systems (Promega) were used, respectively. Cells were seeded at a concentration of 8 x 10^3^ cells per well in a 96-well plate and incubated with spheres or tubes (0.5 mg/mL) for 24 hours. The caspase solution was then directly added to the media to have a 1:1 final ratio, and the luminescence was measured (Varian Cary Eclipse).

## Supporting information

S1 Fig Characterization of tubes and spheres. (**A**) The histograms represent the diameter of the spheres and the relative correlation function measured by DLS. (**B**) Image analysis of Spheres and Tubes from TEM micrographs. Size distribution was measured on 100 nanoparticles for each sample and fitted by a normal Gaussian function using OriginPro8.5 (red line). **(C)** Average morphologic parameters ± standard deviation of spheres or tubes polymersomes. The table clearly shows that the reduction of circularity is dependent on sucrose %.
(DOCX)

S2 Fig Cellular uptake of spheres and tubes in HeLa and HDF. Confocal imaging showing the differential uptake and spatial distribution between spheres and tubes after 96 hours incubation with HeLa or HDF cells. Green is Calcein staining of the cytoplasm.
(DOCX)

S3 Fig Matlab based software for the quantification of NDI and MNi. The software can discriminate cells with one, two, or more nuclei, as well as bi-nucleated cells with a micronucleus.
(DOCX)

S4 Fig (A) GPC chromatogram of PMPC_25_-PDPA_67_ analyzed in DI water + 0.25% (v/v) TFA. (B) ^1^H-NMR spectrum of PMPC_25_-PDPA_67_ in CDCl_3_/CD_3_OD 3:1 (v/v).
(DOCX)
