# Supplementary material for: Tuning cell behavior with nanoparticle shape
Source: PLoS One. 2020 Nov 13;15(11):e0240197. doi: 10.1371/journal.pone.0240197 (PMC7665645; doi:10.1371/journal.pone.0240197)
Supplement: S1 Fig — (A) The histograms represent the diameter of the spheres and the relative correlation function measured by DLS. (B) Image analysis of Spheres and Tubes from TEM micrographs. Size distribution was measured on 100 nanoparticles for each sample and fitted by a normal Gaussian function using OriginPro8.5 (red line). (C) Average morphologic parameters ± standard deviation of spheres or tubes polymersomes. The table clearly shows that the reduction of circularity is dependent on sucrose %. (DOCX) [file pone.0240197.s001.docx]

**S1 Fig. Characterization of tubes and spheres.**

(**A**) The histograms represent the diameter of the spheres and the relative correlation function measured by DLS. (**B**) Image analysis of Spheres and Tubes from TEM micrographs. Size distribution was measured on 100 nanoparticles for each sample and fitted by a normal Gaussian function using OriginPro8.5 (red line). **(C)** Average morphologic parameters ± standard deviation of spheres or tubes polymersomes. The table clearly shows that the reduction of circularity is dependent on sucrose %.
