# Supplementary material for: Tuning cell behavior with nanoparticle shape
Source: PLoS One. 2020 Nov 13;15(11):e0240197. doi: 10.1371/journal.pone.0240197 (PMC7665645; doi:10.1371/journal.pone.0240197)
Supplement: S2 Fig — Confocal imaging showing the differential uptake and spatial distribution between spheres and tubes after 96 hours incubation with HeLa or HDF cells. Green is Calcein staining of the cytoplasm. (DOCX) [file pone.0240197.s002.docx]

**S2 Fig. Cellular uptake of spheres and tubes in HeLa and HDF.**

Confocal imaging showing the differential uptake and spatial distribution between spheres and tubes after 96 hours incubation with HeLa or HDF cells. Green is Calcein staining of the cytoplasm.
