# Supplementary material for: Tuning cell behavior with nanoparticle shape
Source: PLoS One. 2020 Nov 13;15(11):e0240197. doi: 10.1371/journal.pone.0240197 (PMC7665645; doi:10.1371/journal.pone.0240197)

**S3 Fig. Matlab based software for the quantification of NDI and MNi.**

The software can discriminate cells with one, two, or more nuclei, as well as bi-nucleated cells with a micronucleus.


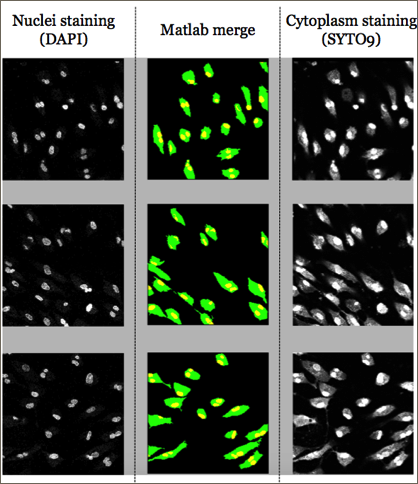

Supplement: S3 Fig — The software can discriminate cells with one, two, or more nuclei, as well as bi-nucleated cells with a micronucleus. (DOCX) [file pone.0240197.s003.docx]
