# Supplementary material for: Tuning cell behavior with nanoparticle shape
Source: PLoS One. 2020 Nov 13;15(11):e0240197. doi: 10.1371/journal.pone.0240197 (PMC7665645; doi:10.1371/journal.pone.0240197)
Supplement: S4 Fig — (A) GPC chromatogram of PMPC25-PDPA67 analyzed in DI water + 0.25% (v/v) TFA. (B) 1H-NMR spectrum of PMPC25-PDPA67 in CDCl3/CD3OD 3:1 (v/v). (DOCX) [file pone.0240197.s004.docx]

**S4 Fig. (A)** GPC chromatogram of PMPC_25_-PDPA_67_ analyzed in DI water + 0.25% (v/v) TFA. **(B)** ^1^H-NMR spectrum of PMPC_25_-PDPA_67_ in CDCl_3_/CD_3_OD 3:1 (v/v).
